# A socio-hydro-epidemiological model for simulating trade-off between dengue infections, water shortages, and adaptive behavior

**DOI:** 10.1016/j.isci.2026.116310

**Published:** 2026-06-08

**Authors:** Maurizio Mazzoleni, Francesco Defilippo, Carlo Torti, Eugenia Quiros-Roldan, Elena Raffetti

**Affiliations:** 1 Institute for Environmental Studies, Vrije Universiteit Amsterdam, Amsterdam, the Netherlands; 2 Istituto Zooprofilattico Sperimentale della Lombardia e dell’Emilia-Romagna “Bruno Ubertini” (IZSLER), Brescia, Italy; 3 Dipartimento di Sicurezza e Bioetica – Sezione di Malattie Infettive, Università Cattolica del Sacro Cuore, Rome, Italy; 4 Dipartimento di Scienze Mediche e Chirurgiche, U.O.C. Malattie Infettive, Fondazione Policlinico “A. Gemelli” I.R.C.C.S., Roma, Italy; 5 Department of Clinical and Experimental Sciences, Unit of Infectious and Tropical Diseases, University of Brescia and ASST Spedali Civili di Brescia, 25123 Brescia, Italy; 6 Unit of Infectious and Tropical Diseases, ASST Spedali Civili di Brescia, 25123 Brescia, Italy; 7 Department of Global Public Health, Karolinska Institutet, Stockholm, Sweden; 8 Swedish Centre for Impacts of Climate Extremes (climes), Uppsala University, Uppsala, Sweden; 9 British Heart Foundation Cardiovascular Epidemiology Unit, University of Cambridge, Cambridge, UK; 10 Victor Phillip Dahdaleh Heart and Lung Research Institute, University of Cambridge, Cambridge, UK

**Keywords:** environmental science, environmental health

## Abstract

Dengue and drought severity are rising worldwide, with drought responses shaping mosquito breeding conditions in cities. Current modeling approaches do not couple household water-use behavior, climate extremes, and vector-borne disease transmission. Here, we developed a system dynamics model that links dengue transmission, human-water interactions, multiple adaptation strategies, and social behavior in a synthetic city. We compared three adaptation pathways: dengue-focused, drought-focused, and co-adaptation guided by social awareness. Results show that adaptation choices strongly affect awareness, water shortages, mosquito abundance, and human infections. Drought-focused adaptation reduces average water shortages, but prolonged standing water in rainwater tanks increases mosquito growth and dengue transmission. Co-adaptation retains drought buffering while limiting favorable habitat for vector growth. Changes in drought-awareness decay can influence dengue outbreaks more strongly than changes in dengue-awareness decay. These results highlight the value of coordinated drought and dengue management for reducing health risks while maintaining water security under climate change.

## Highlights

• A system dynamics model coupling dengue transmission, adaptation, and human-water behavior
• Drought-focused adaptation lowers shortages but can raise dengue risk
• Co-adaptation balances water security and dengue control across climates
• Drought-awareness decay affects dengue outbreaks more than dengue-awareness decay

## Graphical abstract

**Figure:**
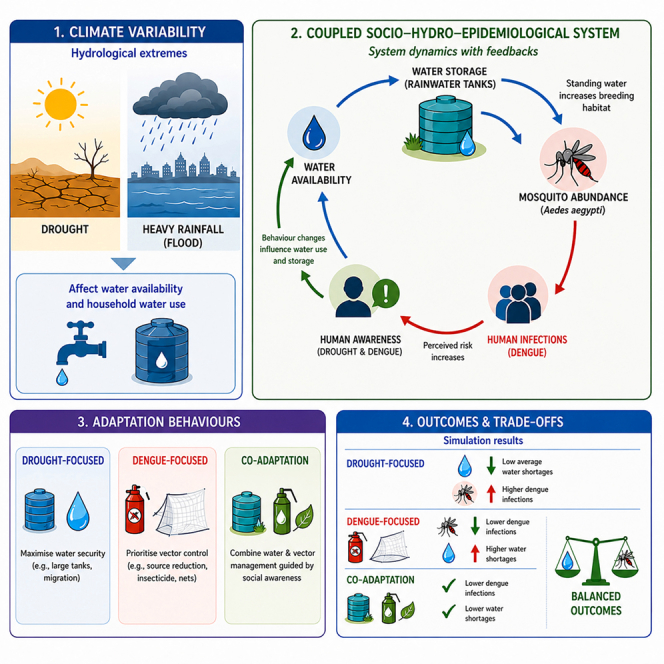

## Introduction

Dengue is a febrile disease (DENF) caused by infection with one of four dengue viruses and it has emerged as the world’s fastest-spreading arboviral disease, with incidence drastically increased between 2000 and 2024.^1^^,^^2^^,^^3^ During the first four months of 2024 alone, the World Health Organization (WHO) recorded 7.6 million reported cases and over 3,000 deaths across 90 countries, already surpassing the global totals for 2022.^4^ Aedes aegypti, the principal DENF vector, have expanded their geographic range into temperate latitudes, high-altitude cities, and peri-urban landscapes once considered inhospitable.^5^

Climatic factors influence DENF mosquitoes distribution and DENF transmission.^6^ The emergence of DENF outbreaks is expected to increase in the future due to climate change.^7^ Temperature and precipitation can strongly shape the availability, quality, and spatial distribution of Aedes breeding sites.^8^^,^^9^ Heavy precipitation can initially flushes out or dilutes container habitats, reducing larval densities, but subsequent ponding in discarded tyres, gutters and construction sites expands larval habitat after a 2- to 4-week lag.^10^^,^^11^ Precipitation deficit can also affect mosquitoes distribution in different ways: (1) moving bodies of water drying up into many still pools, which could serve as vector breeding grounds^12^; (2) increasing the concentration of organic matter in existing containers, improving larval food quality^13^; (3) shifting the vector species dominance favoring Aedes aegypti over Aedes albopictus in drier urban microclimates^14^; and (4) implement behavioral adaptation (particularly household water storage) that proliferates artificial breeding sites.^15^

Climate is only one of many important drivers that influence the transmission, distribution, and incidence of DENF.^16^^,^^17^^,^^18^ Urbanization favors the proliferation of different Aedes mosquitoes. The socioeconomic, cultural, and institutional characteristics of the urban ecosystem play a crucial role in shaping the risk of DENF, human contact with infected vectors, and susceptibility to diseases.^19^ Land-use changes can influence Aedes mosquitoes circulation as irrigated croplands, fragmented forests, and standing water bodies increase the risk of vector-borne outbreaks in Europe.^20^ Human awareness and behavior (e.g., staying indoor during peak mosquito-biting times) can also strongly influence the exposure to mosquitoes and DENF virus transmission.^2^ Different societal vulnerabilities can worsen the impacts of DENF, as they do not affect all individuals equally.^19^^,^^21^^,^^22^ Low-income groups are more at risk of contracting DENF as they may struggle more to prevent mosquito infections using direct methods, such as sprays and repellents.^23^^,^^24^^,^^25^

Human adaptation to hydrological extremes may either amplify or dampen DENF risk in urban areas. In regions where piped water is intermittent or unreliable, households can respond to drought by installing rainwater harvesting tanks, underground cisterns, or plastic drums.^26^^,^^27^ Unless tightly sealed and routinely cleaned, these containers become ideal larval habitats as nutrient-rich and predator-poor.^28^ Studies across Asia showed that household water management and type of water-storage containers, rather than socio-demographic factors, had a strong influence on the density of mosquito larvae.^15^^,^^29^ In Brazil, DENF incidence following extreme drought was higher in highly urbanized areas that had a higher frequency of water supply shortages.^12^ Comparable dynamics have been documented in Vietnam, showing that municipalities with continuous piped-water coverage reduced the probability of DENF under moderate drought conditions than municipalities with poorer coverage.^16^ Moreover, the effect of drought adaptation on DENF can be heterogeneous. A number of studies showed how higher-income households can mitigate risk by investing in sealed tanks, chlorination and routine maintenance,^30^^,^^31^ whereas low-income settlements rely on improvised containers that can exacerbate DENF spread.

Epidemiological compartment models, typically susceptible, exposed, and infected (SEI) models, are widely used to examine how climate and socio-economic conditions shape DENF dynamics and infections.^32^^,^^33^ Seasonal or weather-dependent parameters capture temperature effects on mosquito survival, biting, and the extrinsic incubation period, and rainfall effects on aquatic habitat via flushing, drying, and subsequent pooling. For example, Huber et al.^34^ incorporated thermal response functions for all vector and parasite traits into a dynamic disease transmission model for dengue virus under seasonally varying temperatures. A similar approach was proposed by Alves et al.^35^ to explore the role of climate fluctuations on DENF dynamics from 2010 to 2019 in four Brazilian municipalities. Simple vector-host models were fitted to Singapore to reproduce seasonal fluctuations in vector density using DENF incidence.^36^^,^^37^

Different epidemiological models have been developed to account for the socio-economic conditions (exposure, habitat, and mobility pathways) on DENF infections. Recently, Raj et al.^38^ presented a DENF transmission model for a population with varied age groups, including factors such as age distribution, vector control measures, human knowledge of self-defense, and the latent delay for both humans and vectors. Gholami et al.^39^ developed a model that simulates the dynamics of DENF transmission together with the economic and social burden of the disease in Nepal. Including human mobility within epidemiological models (using for example network couplings) can better explain the spatial spread of DENF. For example, Enduri and Joland^40^ developed an SEIR-SEI model with diffusive human mobility as a reference; the results showed that while human mobility makes the infection spread faster, there is an apparent early suppression of the epidemic compared to immobile humans. Control and adaptation strategies have also been included in epidemiological models to quantify realistic coverage/cadence on the spread of DENF. Tang et al.^41^ proposed a model to closely mimic the integrated program of impulsive vector control and continuous patient treatment and isolation implemented in the Guangdong Province, China, for the 2014 DENF outbreak. A similar DENF transmission model was developed in the study by Luz et al.,^42^ to assess the evolution of insecticide resistance and immunity in the human population, including a cost-effectiveness analysis of different insecticide-based vector control strategies. The results showed that larval control can be counterproductive and lead to epidemics in later years due to changes in insecticide resistance and loss of herd immunity. A comprehensive systematic review of mathematical DENF models is provided in the study by Ogunlade et al.,^43^

System dynamics (SD) models have also been used in hydrological studies as they allow for simulating the link between climate, water use and supply, social behavior, and drought adaptation. Those models represent reservoirs and other water supply systems as “stocks” influenced by climate and withdrawals, while policies (e.g., hedging, conservation campaigns, tariff changes) feedback on social awareness that can influence water use.^44^^,^^45^ Garcia et al.^46^ explored the interactions between water supply, demand, and social awareness of water shortages for various reservoir release policies. Recently, Savelli et al.^47^ developed an SD model to quantify the effect of social inequalities in shaping water shortages in cities during drought events. Reviews show that SD models are suited to stress-testing adaptation portfolios (e.g., conservation, reuse, and rainwater harvesting) under changing climatic conditions and to represent social responses influenced by diverse risk perceptions and behaviors.^48^^,^^49^^,^^50^

Despite considerable progress in modeling DENF and drought occurrence, there are still no epidemiological or SD models that represent the compound effect of droughts, drought adaptation, social behavior, and the mosquito-human transmission cycle leading to DENF and water shortage. Most of the proposed epidemiological models simplify drought as an exogenous shock or neglect drought adaptation pathways, thereby underestimating the reinforcing feedback by which water-storage responses amplify vector breeding. On the other hand, hydrological SD do not account for the epidemiological dynamics and infection assessment.

For these reasons, this study aims to develop a socio-hydro-epidemiological model to simulate the dynamics between climate, society, and vectors to explore how adaptation behaviors can exacerbate the impacts of drought and DENF. The proposed framework will include societal awareness as a key driver for representing adaptation behavior to either drought or mosquito control measures. Different scenarios of behaviors are assumed in this explorative study that is aimed as a transferable modeling framework rather than a site-specific predictive model.

**Figure 1:**
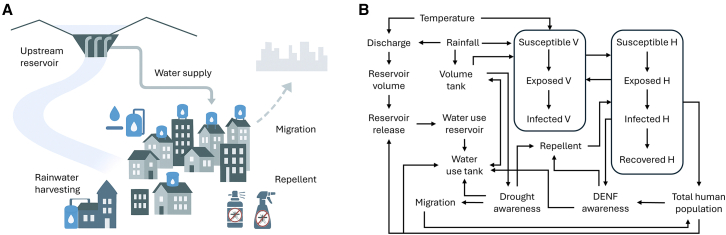
Conceptual schematization of the socio-hydro-epidemiological model (A) Schematic representation of the synthetic case study to explore the dynamics between climate, society, DENF, and adaptation. (B) Causal loop diagram representing the feedback between the modeled variables, with H indicating human, while V the vector.

We combined a parameterized and validated epidemiological model for DENF infection^51^ with an established socio-hydrological model^52^^,^^53^ representing the effect of human behavior on water use and the implementation of different adaptation strategies. We used a system dynamics modeling framework to simulate the dynamics between DENF, climate, society, mosquito growth, and different drought and DENF adaptation (Figure 1B). In this simulation, society can cope with drought and DENF by (1) installing or enlarging rainwater harvesting against drought, (2) migrating to areas less affected by drought, and (3) using more repellent against mosquitoes.

We assumed that changes in societal awareness drive the adoption of a particular management strategy. This will allow us to explore how changes in drought awareness could lead to higher DENF, and how changes in DENF awareness may lead to lower water availability. Based on these adaptations, we assumed different behaviors of risk adaptation as a function of the awareness of each household: (1) DENF-focused behavior with adaptation actions are focused on DENF, (2) Drought-focused behavior, all the adaptation strategies are toward the reduction of water shortages, (3) Co-adaptation behavior, the household can decide to either focus on reducing drought or DENF impacts based on their different awareness values over time.

We apply the model to a synthetic case study (Figure 1A) to: (1) identify causal effects, such as whether increased rainwater tank use and greater public awareness reduce (or exacerbate) DENF outbreaks; (2) run different scenarios of changing climate (e.g., drought-vs. flood-dominant climates) and adaptation behaviors; and (3) avoid overfitting to specific water-supply and socioeconomic contexts. In particular, with this approach, we want to explore the benefit and harm of different intervention polices across contrasting climate extremes to identify “no-regrets” combinations. More information about the model structure and equations is available in the STAR Methods section.

### Science for society

This study proposed the first socio-hydro-epidemiological model that simulates household drought adaptation, awareness, and dengue transmission within a system-dynamics framework.

The findings reveal a strong dependence between adaptation behaviors, water shortages, and dengue infections. Single-hazard strategies can backfire: prioritizing water security without steady mosquito control practices raises dengue infections, while dengue-first strategies can worsen shortages. Co-adaptation strategy mitigates this trade-off and performs robustly across drought- and flood-dominant climates. The findings of our study show that changes in drought awareness decay have a stronger influence on dengue infection outbreaks than decay in dengue awareness in both drought- and flood-dominant climates.

Our modeling approach can be used for: (1) testing hypotheses about how drought and dengue risks are generated by the interaction between consecutive hydrological extremes and human behavior; and (2) suggesting additional types of observations to monitor the dynamics between climate, society, and dengue infections. These insights can help policymakers in the exploration of adaptation pathways and trade-offs under global anthropogenic and climatic changes based on an advanced general understanding of the socio-hydro-epidemiological dynamics. The model is published open access and can be customized to meet the needs of different end-users.

## Results

### Effect of risk adaptation behaviors on water shortages and DENF

**Figure 2:**
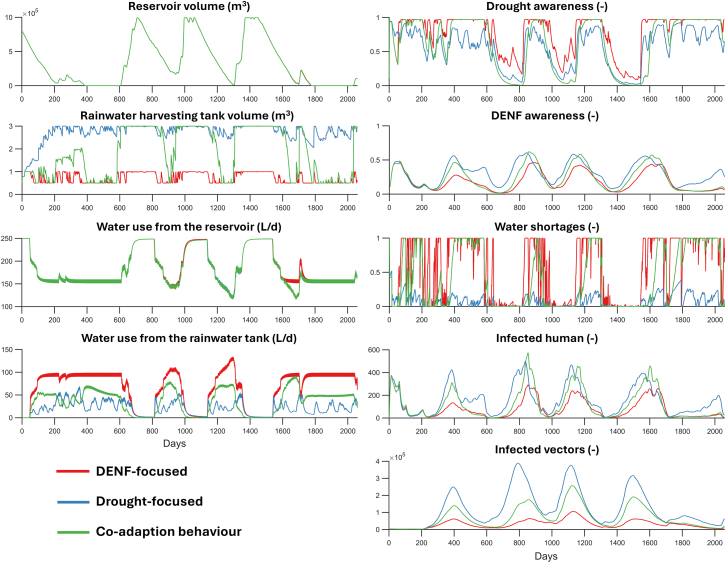
Predictive modeling results Time series of the main model output during the drought climate scenario in response to three different adaptation behaviors.

In this analysis, we focus on the effect of different risk adaptation behaviors and unintended consequences when coping with water shortages and DENF. Overall, the results show how adaptation behaviors strongly influence the evolution of awareness, mosquito abundance, and DENF infections. We can observe that when society adopted drought-focused measures, the water shortages decrease, while the number of infected humans increases. On the other hand, when focusing on DENF measures, infected people reduce but increase water shortages (Figure 2). When a co-adaptation behavior is present, a trade-off between reducing drought and DENF impact is achieved.

Climate strongly modulates reservoir and tanks storage: extended wet spells keep household tanks near capacity, while dry spells trigger rapid drawdowns. Water use from the reservoir shows a similar pattern. Households then shift water supply sources and adapt use according to the restrictions in section 2.2.1, with drought awareness declining as tank volume rises. The three adaptation behaviors show different water use and water availability patterns in response to hydroclimatic variability. The drought-focused behavior maintains full tanks for long periods, followed by abrupt depletion during drought. The DENF-focused behavior intentionally keeps lower volumes—avoiding capacity increases in dry periods to limit mosquito breeding—so water use changes more and tanks empty faster. The co-adaptation behavior shows frequent, partial water reduction and refills even with normal levels of the reservoir. These differences in adaptation behavior on water availability directly affect drought and DENF awareness and losses.

Drought-oriented behavior shows lower values of drought awareness compared to the other two behaviors due to the implementation of drought measurement that can guarantee water availability and reduce water shortages. A rapid increase follows an abrupt tank depletion around day 900 in drought awareness. A similar pattern occurs around day 1200 with awareness increasing when the reservoir volume drops to then reduces again due to the full rainwater harvesting tank. In the DENF-oriented and adaptive behavior, drought awareness shows higher values and variability over time as influenced by rapidly changing water shortages. DENF awareness is overall higher in drought-oriented adaptation due to a higher number of infected individuals. On the other hand, DENF-oriented and adaptation behavior tend to show similar patterns, highlighting the importance of accounting for both drought and DENF impact in the decision-making process.

Infection outbreak timing is similar across adaptation behaviors, showing similar influences of climate forcing, dry and wet periods, and mosquito-human feedbacks, but with different amplitudes. In particular, higher infection peaks occur in the transition between dry to wet conditions, especially when the rainwater tanks are kept full for a long period. The drought-oriented behavior produces the highest values in both infected vectors and humans, including a late epidemic that substantially exceeds the outbreaks from the other two adaptive behaviors. The DENF-oriented pathway consistently shows lower infections, due to the fact that tanks stay full for short periods and apply mosquito control measures more continuously. Similar patterns can be observed in the co-adaptation behavior, with lower infections during dry periods (e.g., between days 900 and 1100) but higher peaks that occur after the ones for the drought-oriented behavior.

### Effect of hydrological extremes

**Figure 3:**
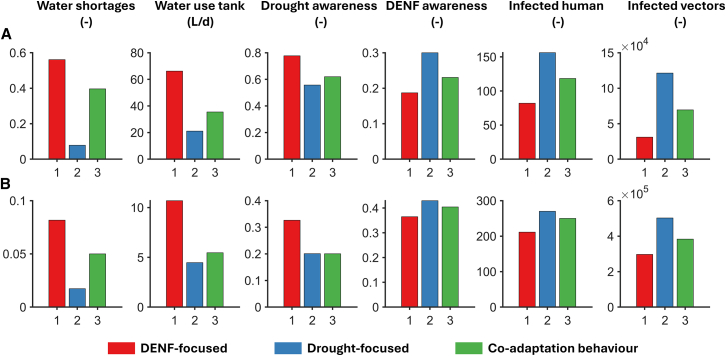
Summary of the model results for different climate scenarios Average value of different model results in case of drought climate (A) or flood climate (B) scenarios and three adaptation behaviors.

Here, we assess model results when forced by climate scenarios with the prevalence of drought periods (Figure 3A) and flood periods (Figure 3B). The aggregated indicators reveal that hydroclimatic conditions can substantially affect the coupled dynamics of drought and DENF virus transmission, while the results of for the three adaptation behaviors remain consistent.

In the drought climate scenario (Figure 3A) water shortages are a recurrent challenge. Here, a society with drought-focused behavior experiences the lowest cumulative water shortages, confirming the importance of water storage in mitigating scarcity. DENF-focused behavior, by contrast, shows the highest water shortages as society keeps lower storage volumes to avoid mosquito breeding. Co-adaptation behavior achieves lower water shortages than DENF-focused behavior, highlighting the importance of the trade-offs between behaviors. Water use from the tank and drought awareness follow a similar pattern. A drought-focused behavior society uses the least from tanks because their storage is largely retained to buffer future water shortages, whereas DENF-focused behavior relies more heavily on tank water. Co-adaptation behavior displays intermediate tank use. The awareness results show that prolonged full-tank conditions under drought-focused behavior lead to lower drought awareness, while we found a slightly higher awareness in the co-adaptation behavior due to the adaptive tank cycling management. Opposite results are found for the DENF awareness; the highest values are found in drought-focused behavior, reflecting reactive responses to infection outbreaks, while showing lower values for the other adaptation behaviors. Epidemiological outcomes diverge significantly in the drought-dominant climate. Drought-focused behavior experiences the highest vector and human infections, as long-standing full tanks provide persistent breeding habitat. DENF-focused behavior achieves a substantial reduction in both human and vector infections, but at the cost of significantly higher water shortages. As in the case of water shortages, co-adaptation behavior provides an intermediate number of human and mosquito infections if compared to the other two behaviors, highlighting the potential of adaptive strategies again.

The flood climate scenario (Figure 3B) shows comparable trends but different intensity of the drought and DENF losses. On the one hand, frequent and intense rainfall events (Figure 3B) reduce water shortages for all adaptation behavior, narrowing the gap between behavioral strategies. On the other hand, the wet periods produce more breeding habitat, which increases DENF risk. DENF awareness rises in every adaptation behavior, as households encounter more frequent signs of vector presence or disease. Under flood conditions, different adaptation behaviors matter less, with the co-adaptation behavior remaining the most efficient compromise.

### Effect of drought and DENF awareness decay with adaptive risk attitude

**Figure 4:**
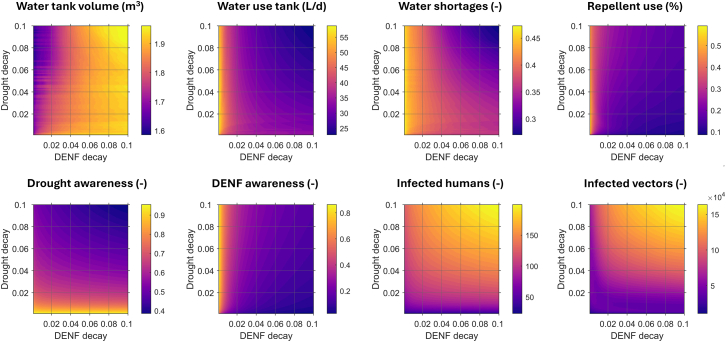
Sensitivity analysis for awareness decay, drought climate scenario Average value of different model results for different combinations of drought and DENF awareness decay in the case of drought climate scenario.

This analysis explores the effect of drought and DENF awareness decay on drought and DENF impacts, considering the drought climate scenario. The results in Figure 4 show the average value of the model outputs for different combinations of drought and DENF awareness decay. A lower value of awareness decay means that awareness will fade slowly, while a higher decay values correspond to rapid forgetting. The findings of this analysis show that changes in drought awareness decay have a stronger influence on DENF outbreaks than decay in DENF awareness. Changing values of drought decay provides similar higher values of water use from the rainwater harvesting tank, while increasing DENF awareness decay leads to lower water use. The lowest values of water use from the rain tank are observed for the highest values of both drought and DENF decay, while the highest values are observed for low DENF decay. Opposite patterns are found for the volume of the rainwater harvesting tank, where the highest values correspond to high DENF awareness decay, while the lowest correspond to high water use values. Water tank volume decreases for high drought awareness decay, as when society tends to forget faster about drought, they tend to use more water and empty the tank faster. Water shortages show a similar pattern to water tank volume, with high loss values found with low DENF awareness decay and low losses with both high drought and DENF decay. The percentage of repellent use shows overall small values around 10%, lower than the minimum repellent use if DENF awareness is higher than drought awareness, with higher percentages with low DENF decay as society that tend to forget slower about DENF will use more repellent. As expected, the results highlight a strong dependency between changes in drought decay and drought awareness. Higher values of drought awareness are found for low drought decay, as a society that forgets slower water shortages shows higher and persistent drought awareness. DENF decay does not strongly affect drought awareness. Similarly, drought decay does not influence DENF awareness, and high values are found for low DENF decays.

The infection results reveal two main patterns. A storage-dominant area (slow drought decay and fast DENF decay) shows elevated mosquito and human infections, reflecting high volume of rainwater tanks with weak personal protection. A protection-dominant quadrant (fast drought-decay and slow DENF-decay) shows reduced infections for both mosquitoes and humans due to strong repellent use and restrained tank volume. Where both drought and DENF are quickly forgotten (high decay values), infections remain moderately high: repellent use is weak and rainwater tank volume does not compensate. Where both awareness persist (low decay), the results indicate lower infections. We also found that small changes in either awareness decay can shift the system between high- and low-infection regions.

Across both infection panels, outcomes vary systematically with the decay of drought and DENF awareness. For constant DENF decay values, increasing drought awareness decay tends to increase the volume of rainwater harvesting. This results in higher water availability, and higher mosquito and human infections are found when both drought and DENF awareness decay fast. Varying DENF awareness decay while keeping constant drought awareness decay produces the complementary effect. As DENF awareness decays more slowly, repellent use increases, and rainwater harvesting tank volume is low. In the vector infection panel, this corresponds to a broad region of lower mosquito infections, with the lowest levels occurring where DENF awareness is strong, regardless of drought awareness. The human infection panel mirrors this pattern: human infections decline with sustained DENF awareness and are lowest where repellent uptake is high. Where DENF awareness decays rapidly (fast forgetting), both panels show higher infections, especially when compared with persistent drought awareness. Similar results have been found for the flood climate scenario and are included in the supplemental information (Figure S1).

### Model sensitivity analysis

**Figure 5:**
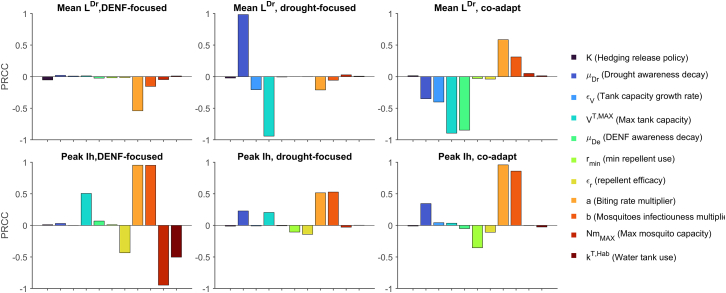
Sensitivity analysis for model parameters, drought climate scenario Results of the model sensitivity analysis with L^Dr^ water shortages and Ih number of infected humans in case of drought climate scenario.

We quantified the influence of model parameters on the mean water shortages (*L*^Dr^) and the peak number of infected humans (*Ih*) using a global sensitivity analysis based on latin hypercube sampling (LHS) combined with partial rank correlation coefficients (PRCC). PRCC was computed separately for each adaptation behavior using rank-transformed inputs and outputs. Figure 5 shows that a small subset of parameters influences both outcomes, while most parameters show PRCC values close to zero, indicating limited influence within the explored ranges.

For the mean water shortages, the model results are very dependent on the risk behavior. The drought-focused behavior is strongly influenced by drought and DENF awareness and reservoir-tank parameters (*ε*_V_ and *V*^T,MAX^), with larger maximum tank capacity reducing shortage (negative PRCC). In the co-adapt behavior, the mean water shortage is most sensitive to the persistence of both drought and DENF-related responses (*μ*_Dr_, *μ*_De_) and to reservoir-tank parameters with pronounced negative PRCCs, implying that these parameters are associated with lower shortage levels under coupled decision-making. Mosquito biting rate and infectiousness have a positive link with mean shortages. In contrast, the DENF-focused behavior shows weaker and mixed sensitivities for shortage, with mosquito biting rate and infectiousness influential (negative PRCC), while other parameters produce small effects. For peak infected humans (*Ih*), the mosquitoes’ characteristics (mosquito biting rate, infectiousness, and maximum capacity) are consistently among the strongest drivers across risk behaviors (positive PRCC). In DENF behavior, *N*_mMAX_ shows a negative PRCC, implying that a larger mosquito carrying capacity is associated with smaller epidemic peaks and likely reflects nonlinear feedbacks in the coupled system (e.g., earlier outbreaks and faster susceptible depletion). In the DENF-focused and co-adaptation behaviors, repellent-related parameters (minimum use *r*_min_ and efficacy *ε*_r_) contribute with negative PRCC values, consistent with reduced transmission under stronger protection. With co-adaptation behavior, sensitivity additionally involves the links between storage and habitat (*k*^T,Hab^), repellent use, and mosquito capacity terms, highlighting the role of coupled drought-dengue feedbacks. Similar analyses were performed for the flood climate scenario, and results are reported in the supplemental information (Figure S2).

The results of the uncertainty analysis (Figures S3 and S4) show the Monte Carlo distributions of peak *Ih* and mean shortage under the same global sampling of uncertain parameters. The three risk behaviors generate distinct distribution shapes, implying that behavioral assumptions influence not only central tendencies but also the spread and multimodality of model results, depending on how behaviors interact with hydrologic and epidemiologic processes.

## Discussion

This study proposed the first socio-hydro-epidemiological model that simulates household drought adaptation, awareness, and dengue transmission within a system-dynamics framework. Our results highlight the close relationship between hydro-climatic variability, water shortages, household water management, risk of DENF, and adaptive behavior. In particular, under drought-dominant conditions, our findings identify the potential for maladaptation as rapid expansion of rainwater harvesting tanks without repellent protection can lead to an increase in DENF once rains resume. Our simulation highlights the benefits of co-adaptation strategies to reduce DENF and maladaptation from strategies focused on keeping tanks full during drought without sustained dengue practices. We show that when rainwater storage becomes central to drought adaptation, the stagnant water availability governs a large share of urban Aedes aegypti breeding sites. In line with those findings, our simulation highlights that drought (mal) adaption strategies that keep tanks full without sustained dengue practices yield the highest vector and human infections, whereas pairing storage with basic protection suppresses averages and narrows variability. This relationship has been widely documented, where piped supply is unreliable, tanks and cisterns sustain vectors through dry seasons, and transmission occurs when favorable climatic conditions occur.^54^^,^^55^ Field studies in Australia and Brazil identify poorly screened tanks and large containers as frequent larval sources, particularly through dry seasons.^56^

Climate modulates is a key driver of both drought and DENF risks: under drought-dominant conditions, storage is the primary habitat and behavioral choices loom large; under flood-dominant conditions, intense rainfall supplies abundant ephemeral breeding sites that increase DENF risks even for societies that combine vector-safe rainwater tanks with personal protection.^5^^,^^12^^,^^57^^,^^58^^,^^59^ In flood-dominant climates, we found that behavioral differences persist but are limited, as climate itself creates abundant larval habitat. This aligns with studies linking post-flood periods to dengue outbreaks, as intense precipitation events generate widespread ephemeral breeding sites, reducing the relative importance of household storage.^60^^,^^61^ In such climates, preventive strategies must combine environmental management during wet periods with storage security during inter-flood intervals to sustain protection.

Concerning different adaption behaviors, we found that society tends to undertake preventive actions only when the impact is high; once cases fall and the threat feels distant, adaptation reduces.^62^^,^^63^ Because preventive action often starts late, outbreaks have time to grow before control can be effective, which leads to large infection peaks. Our results, together with work in socio-hydrology, point to a common driver behind this pattern: risk awareness critically shapes adaptive behavior. We show that as awareness changes, society can shift between three broad modes: “storage-dominant”, heavy reliance on rain storage after a major drought as drought awareness is high^64^^,^^65^; “protection-dominant”, steady personal protection after a disease outbreak due to high DENF awareness,^66^ or a mix of both. The worst outcomes arise when both drought and DENF awareness decrease quickly: households refill and keep tanks just as vigilance drops, so mosquito populations and infections increase together. By contrast, keeping at least one awareness strong stabilizes the system. If DENF awareness lasts, people maintain personal protection and transmission stays low even with high storage.^67^^,^^68^ If drought awareness lasts, households keep storage lower and reduce habitat even when dengue prevention are not adopted; this could be achieved by adopting campaigns that remind people about past water shortages.^69^

The results of the sensitivity analysis indicate that the dominant controls on system performance are behavior-dependent. The mean water shortage is primarily influenced by risk behavior and tank storage dynamics, with larger tank capacity associated with reduced shortage and faster loss of drought awareness generally associated with higher shortage. For epidemic intensity, drought-focused behavior can indirectly affect DENF transmission via storage-related pathways.^70^ Repellent parameters show negative PRCC values, consistent with exposure reduction lowering transmission peaks. Parameters linked to mosquitoes’ characteristics (biting rate, infectiousness, and maximum capacity) are the ones mainly affecting model output. The relevance of the storage-habitat coupling term (*k*^T,Hab^) under co-adaptation behavior is also consistent with evidence that rainwater storage systems can provide or sustain Aedes habitat when not effectively managed.^71^

The strength of this model lies in the explicit representation of the effect of climate adaptation strategies on an opposite hazard such as DENF infections. In previous studies, these two were modeled independently, while their coupling allowed us to identify trade-offs and unintended adaptation consequences. Moreover, the synthetic setting of this study allowed us to explore the climate-human-vector mechanisms under different hypothetical scenarios of adaptation behaviors and climate, assessing how rainwater tank residence time and the decay of drought and DENF awareness shape vector capacity and DENV infections. The qualitative model output pattern is robust and matches observations in many settings: storage-focused strategies trade lower drought loss for higher dengue burden,^70^ protection-heavy strategies do the opposite,^72^ adaptive strategies capture most benefits with fewer downsides.^73^

The findings also carry clear governance implications. First, vector-proof design must be standard for all domestic storage, particularly in regions prone to climate extremes. Inexpensive measures, screened inlets, first-flush diverters, and drain valves, can convert potential vector habitats into safe drought assets.^71^^,^^73^ Second, on the behavior side, short, seasonal reminders tied to climate outlooks and neighborhood mosquito indices help households maintain container hygiene and use repellent without causing fatigue.^74^^,^^75^^,^^76^ Linking early-warning systems, such as ENSO-based seasonal forecasts or neighborhood larval indices, to SMS reminders or local radio campaigns can activate pre-emptive household action before epidemic peaks. Third, cross-sectoral accountability is essential. While water agencies promote storage to buffer supply fluctuations, health departments bear the cost of resulting vector proliferation. Joint metrics, such as liters of mosquito-proof volume of rainwater tanks per capita and community-level Breteau indices as proxy of density of mosquito breeding sites, can help align incentives and foster co-managed adaptation.^77^ Finally, migration can reduce local demand when drought is severe, but it may shift risk elsewhere; robust systems lower the need for mobility by making in-place adaptation effective.^78^

### Limitations of the study

Our model is also inevitably affected by limitations. First of all, this is an exploratory study aimed at developing a transferable model of the climate-socio-epidemiological dynamics and trade-offs rather than a site-specific predictive model. The model’s robustness has been tested, but empirical calibration against observed dengue cases, Aedes indices, or household storage practices needs to be carried out if applied to a specific case study with similar epidemiological and water management characteristics. We use simplified “awareness” variables and aggregated outcomes; it does not show event timing, social spread of information, housing differences, or micro-climates. Moreover, the model does not represent specific local social, economic, regulatory, and educational contexts that vary substantially worldwide. Future work should focus on applying the proposed model to a real case study and calibrating the model with local data, such as the number, distribution, and seasonality of infected humans and mosquitoes, rainwater tank volume and coverage, and behavior surveys. Our model should be complemented by empirical research and instrumental case studies to advance theory and better understand how the interactions and feedback mechanisms between physical and social processes influence drought and DENF. The model could be extended to include social learning networks and test policy portfolios that combine design standards, inspection frequencies, and timed reminders. We did not explicitly considered for the effect of dry to wet transitions, but wet and dry spells alternate within each scenario and that outbreak timing in the model is sensitive to dry to wet transitions in the epidemiological model. Despite the caveats, the results and governance implications are consistent: vector-safe storage plus steady personal protection, coordinated across water and health institutions, produces lower and more stable dengue burden while preserving drought resilience in both dry- and flood-leaning climates.

## Resource availability

### Lead contact

Further information and requests for resources should be directed to and will be fulfilled by the lead contact, Dr. Maurizio Mazzoleni (m.mazzoleni@vu.nl).

### Materials availability

This study did not generate new unique reagents.

### Data and code availability

• Data: The data reported in this paper are available at Zenodo (https://zenodo.org/records/16934326).
• Code: The code to reproduce the results of this paper is available at Zenodo (https://zenodo.org/records/14917599).
• All other requests: Any additional information is available from the lead contact upon request.

## Acknowledgments

M.M. was supported by the European Union/s Horizon Projects CLIMAAX (grant agreement no. 101093864) and ICISK (grant agreement no 101037293). E.R. was supported by the 10.13039/501100006636Swedish Research Council for Health, Working Life and Welfare (10.13039/501100006636FORTE, grants no. 2022-00882 and 2024-00833), Swedish Research Council for Sustainable Development (10.13039/501100001862Formas, grants no. 2023-01774 and 2022-01845), 10.13039/501100004359Swedish Research Council (10.13039/501100004359VR, grants no. 2023-01982 and 2022-06599).

## Author contributions

Conceptualization, formal analysis, investigation, methodology, and writing – original draft, review and editing, M.M.; writing – original draft, review and editing, F.D., C.T., E.Q.-R., and E.R.

## Declaration of interests

The authors declare no competing interests.

## STAR★Methods

### Key resources table

**Table:** 

REAGENT or RESOURCE	SOURCE	IDENTIFIER
Observed climate and infection data	Caldwell et al. (2021)	https://github.com/jms5151/SEI-SEIR_Arboviruses
Software and algorithms	The code to reproduce our results can be found in Zenodo	https://zenodo.org/records/16934326
MATLAB Software	The MathWorks Inc. (2024). MATLAB version: R2024a, The MathWorks Inc.	https://www.mathworks.com

### Method details

#### Synthetic case

We considered a synthetic urban situation in which DENF can develop in a city water supplied by an upstream reservoir and individual rainwater harvesting, depending on the risk attitude. The synthetic urban area characterised by different climates and social risk behaviour to explore adaptation strategies on water shortages and DENF.

Our recent studies inspire the socio-hydrological component of the model,^52^^,^^53^ while the epidemiological module is based on the one developed, parameterized, and validated in^51^ in 3 different countries. We considered two climate scenarios characterised by a prevalence of either dry (drought) or wet (flood) conditions from the regions of Machala (Ecuador) and Msambweni (Kenya), respectively.^51^ Measurements of temperature, humidity, and rainfall were derived from.^51^

Based on the precipitation and temperature scenario displayed in Figure S5, we used the GR4J hydrological model GR4J^79^ to generate plausible inflow series consistent with the imposed forcing to assess the discharge into the reservoir of the synthetic case study. The GR4J is a lumped rainfall-runoff model that transforms a daily sequence of precipitation and potential evapotranspiration into discharge through two conceptual stores and two unit hydrographs. We set the GR4J parameter values so that the reservoir would have enough water to meet the water-supply demand of the hypothetical population leaving downstream. The GR4J model was not meant to recreate an observed hydrograph, but rather to assess discharge values (and consequent input to the reservoir) from the climatic information from.^51^ An application to a real-world case study should calibrate both GR4J to observed discharge and reservoir operating rules to local practice. We used the Hargreaves–Samani method^80^ to assess evapotranspiration from temperature. GR4J captures key catchment-scale water balance and timing effects while remaining computationally efficient for simulation.

#### Modelling framework

The model is composed of three different systems:

• Reservoir system: It accounts for the water supply, spillway, and required environmental flow released by the reservoir using a linear hedging rule approach. The release for water supply is influenced by the inflow to the reservoir and the downstream water use.
• Households and rainwater harvesting: We calculated the water use based on release from the reservoir and the water availability from the rainwater harvesting system, which is a function of both drought and DENF awareness.
• Epidemiological module: This module simulates DENF as a result of the dynamics between the vector and human population based on an SEI-SEIR model. The model was modified by also including the effect of additional water availability from the rainwater harvesting on the vector growth, as well as the effect of repellent and migration on changes in the total human population.

In each system, both the physical components and the decisions that affect the system were modelled at a daily time scale. In this model, society can cope with drought and DENF (through mosquitoes growth control) by implementing different adaptation strategies. In particular, they can decide to

i Install or enlarge rainwater harvesting to use more water during drought periods
ii Decide to migrate to areas less affected by drought, thus reducing the total population number, after an intense drought period
iii Use more repellent against mosquitoes to reduce DENF risk.

The model ordinary differential equations were solved using an explicit Euler method at a daily time step. All state variables are constrained to remain non-negative, and results are insensitive to initial conditions after a short model warm-up period.

#### Reservoir system module

This module represents the water supply from the upstream reservoir, providing water to the downstream urban area. In particular, we model the water release based on the upstream inflow, downstream water use, environmental flow, spill release during flood conditions, and evaporation from the reservoir. Thus, the variation of reservoir volume *V*^*R*^ over time is assessed as:

(Equation 1) $$\frac{dV^{R}}{dt}=I_{t}^{R}-W_{t}^{R}-S_{t}^{R}-E_{t}$$

where *I*^*R*^ is the mean inflow to the reservoir, *W*^*R*^ is the water withdrawal flow for water supply, *S*^*R*^ is the spillway release flow, and *E* is the environmental flow. We have assumed a maximum value of reservoir volume of 10^6^ m^3^ and an initial volume value equal to 80% of such maximum value. A one-point linear hedging rule^81^ is used to assess the water withdrawal from the reservoir (*W*^R^) for water supply purposes:

(Equation 2) $$W_{t}^{R}=\left\{ \begin{array}{c} \frac{{\;V}_{t}^{R}+I_{t}^{R}\cdot \Delta t-E_{t}\cdot \Delta t}{K}\;\text{if}\;{\;V}_{t}^{R}+I_{t}^{R}\cdot \Delta t-E_{t}\cdot \Delta t\;<KU_{t}^{R}{Nh}_{t}\Delta t\\ 0\;\text{if}\;{\;V}_{t}^{R}\;<0.1\;FSV\\ U_{t}^{R}{Nh}_{t}\;Otherwise \end{array}\right.$$

where *U*^*R*^ is the daily per-capita water use from the reservoir, *K* is the hedging release policy coefficient of the reservoir set equal to 3, and *Nh* is the total population of the downstream area. Once the reservoir volume is higher than the Full Supply Volume (FSV), spillway release occurs, and it is calculated as:

(Equation 3) $$S_{t}^{R}=\left\{ \begin{array}{c} 0\;\\ \left({\;V}_{t}^{R}+I_{t}^{R}\cdot \Delta t-E_{t}\Delta t-FSV\right)\frac{1}{\Delta t}\;\text{if}{\;V}_{t}^{R}+I_{t}^{R}\Delta t-E_{t}\Delta t>KU_{t}^{R}{Nh}_{t}\Delta t+FSV \end{array}\right.$$

The value of FSV is kept constant in this model as we assume that society will cope with drought conditions by implementing individual measures, such as installing and expanding rainwater harvesting tanks, rather than adopting collective measures, such as expanding the upstream reservoir capacity. The evapotranspiration from the reservoir is assessed as:

(Equation 4) $$E_{t}=0.00409\cdot 6.11\cdot e^{\frac{17.3\cdot T_{t}}{237.3+T_{t}}}\cdot A_{res}$$

Where *T* is the temperature and *A*_res_ is the area of the upstream reservoir. The environmental flow is calculated as 5% of the inflow to the reservoir.

During drought conditions, the per-capita water use from the reservoir is reduced to lower the water withdrawals and avoid future critical water shortages. Based on different threshold levels of the reservoir, we associated specific upper and lower values of per-capita water use. Once the reservoir volume is lower than a certain threshold level, the per-capita water use was changed to the corresponding target use *U*^R,^∗ as:

(Equation 5) $$U_{t}^{R,*}=U^{R,U}+\frac{U^{R,U}-U^{R,L}}{V^{U}-V^{L}}\left(V_{t}-V^{U}\right)$$

Where *V*^U^ and *V*^L^ are the upper and lower response triggers, while *U*^R,U^ and *U*^R,L^ are the associated upper and lower target per capita water use from the reservoir.

#### Households and rainwater harvesting module

This module assesses the water supply, water use from the upstream reservoir, and use from the rainwater harvesting system installed in each household as a function of drought and DENF awareness. Based on the target per-capita use, we assess the household’s willingness to reduce water consumption *N*^D^ as:

(Equation 6) $$\frac{dN}{dt}=\left\{ \begin{array}{c} \frac{U^{R}-U^{R,*}}{U^{*}}\left(1-N\right)-\mu_{N}N\Delta t\;\text{if}\;U^{R}>U^{R,*}\\ \mu_{N}N\Delta t\;\text{if}\;U^{R}<U^{R,*} \end{array}\right.$$

Where *μ*_N_ is a parameter representing the decay of the willingness of the household to adopt the target per-capita use over time, set equal to 0.03. As a result, the per-capita use from the reservoir is calculated as

(Equation 7) $$\frac{dU^{R}}{dt}=\left\{ \begin{array}{c} -U^{R}\left[N\alpha_{D}\left(1-\frac{U_{\min}^{R}}{U^{R}}\right)\right]\;\text{if}\;\frac{dN}{dt}>0\\ U^{R}\left[N\alpha_{D}\left(1-\frac{U^{R}}{U_{\mathrm{MAX}}^{R}}\right)\right]\;\text{if}\;\frac{dN}{dt}<0\; \end{array}\right.$$

where *U*^*R*^_min_ is the minimum per-capita water use of 100L per day required for basic health and hygiene, *U*^R^_MAX_ is the maximum per-capita use of 250L per day, while *α*_*D*_ is the parameter representing the decay rate of changes in consumption equal to 0.15.^46^^,^^52^ The volume of water in the rainwater harvesting tank is assessed as:

(Equation 8) $$\frac{dV^{T}}{dt}=\left\{ \begin{array}{c} -V^{T}\;\text{if}\;V^{T}+I^{T}\;<U^{T}\Delta tI^{T}-U^{T}\;\text{if}\;V^{T}+I^{T}\geq U^{T}\Delta t\\ \begin{array}{c} I^{T}-U^{T}\;\text{if}\;V^{T}+I^{T}\;<V^{T,\mathrm{MAX}}\\ 0\;otherwise \end{array} \end{array}\right.$$

Where *I*^T^ is the amount of water flowing into the rainwater tank, calculated as the product of the runoff coefficient, *ϕ* equal to 0.8, the catchment area of the building, *C* assumed equal to 100m^2^, and the precipitation at time *t*. The initial value of the rainwater harvesting tank volume is set equal to half of the maximum capacity.

The water use from the rainwater harvesting tank, *U*^T^, is assessed based on the water use available from the reservoir (which depends on drought conditions and drought release management) and on the drought and DENF awareness at the previous time step. Empirical studies showed that during drought events, society tends to use less water and build bigger storage systems to cope with current and future drought extreme conditions.^46^^,^^82^ In particular, in the case of high DENF awareness, the households will tend to use more water and keep a low tank volume to reduce mosquito spread.

(Equation 9) $$U_{t}^{T}=\left\{ \begin{array}{c} \left({1-A}_{t-1}^{Dr}\right)\left(U_{\mathrm{MAX}}^{R}-U_{t}^{R}\right)\;\text{if}\;\frac{dA^{Dr}}{dt}>\frac{dA^{De}}{dt}\\ \left(U_{\mathrm{MAX}}^{R}-U_{t}^{R}\right)\;\text{if}\;\frac{dA^{Dr}}{dt}<\frac{dA^{De}}{dt} \end{array}\right.$$

We set an initial hypothetical value of water use from the reservoir as 250L per day. Water shortage *L*^DR^ , defined as a dimensionless daily shortage index (0–1), is assessed as a function of the tank volume at time *t*, its minimum (*V*^T,min^) and maximum (*V*^T,MAX^) values, as indirectly affected by the water uses from the tank and reservoirs:

(Equation 10) $$L_{t}^{Dr}=1-\frac{\left(V_{t}^{T}-V^{T,\min}\right)}{\left(V_{t}^{T,\mathrm{MAX}}-V^{T,\min}\right)}$$

Drought and DENF awareness (*A*^*Dr*^ and *A*^*De*^) represent the accumulation of awareness because of the shocks experienced by society during the drought events and DENF infections and their decay due to forgetting experiences.^83^^,^^84^ Drought and DENF awareness are dimensionless, normalised variables bounded between 0 and 1, where 0 indicates no perceived risk, and 1 indicates maximum perceived risk. Variation of drought awareness *A*^*Dr*^ is thus calculated as:

(Equation 11) $$\frac{dA^{Dr}}{dt}=L^{Dr}\left(1-A^{Dr}\right)-\mu_{Dr}A^{Dr}$$

where *μ*_Dr_ is the model parameter representing the decay of drought awareness in time, assumed equal to 0.03. Similarly, the DENF awareness is assessed as a function of DENF losses, expressed as the ratio between infected (*Ih*) and total (*Nh*) population, and the decay of DENF awareness in time *μ*_De_ (0.03):

(Equation 12) $$\frac{dA^{De}}{dt}=\frac{Ih}{Nh}\left(1-A^{De}\right)-\mu_{De}A^{De}$$

Based on the different values of drought and DENF awareness, the households can decide to either increase the maximum volume of the rainwater tank (*V*^T,MAX^) to better fight drought periods or do not change the tank volume to cope with the growth and spread of mosquitoes. We assumed an initial low drought and DENF awareness values of 0.1.

The change in the maximum rainwater tank volume is linked to changes in drought and dengue awareness. In particular, higher drought awareness leads to greater changes in tank volume, while no changes occur if drought awareness changes or is higher than the ones in drought.:

(Equation 13) $$\frac{dV^{T,\mathrm{MAX}}}{dt}=\left\{ \begin{array}{c} A^{Dr}\varepsilon_{V}\left(V^{T}-U^{R}\right)\Delta t\;\text{if}\;\frac{dA^{Dr}}{dt}>\frac{dA^{De}}{dt}\\ 0\;\text{if}\;\frac{dA^{Dr}}{dt}<\frac{dA^{De}}{dt} \end{array}\right.$$

Where *ε*_V_ represents the tank capacity growth rate st equal to 0.01.

#### Epidemiological module

We adapted the SEI-SEIR model developed and validated by^51^ and parameterized for DENF virus transmission in Aedes aegypti mosquitoes as done in.^34^^,^^51^ The model is based on the assumption that mosquito growth is function of temperature,^34^ the mosquito-carrying capacity varies with accumulated 14-day rainfall,^85^ and mosquito mortality varies with saturation vapour pressure deficit (function of temperature and humidity).^86^ The adult vector population (*Nm*) is divided into susceptible (*Sm*), exposed (*Em*), and infected (*Im*) as:

(Equation 14) $$\frac{dSm}{dt}=\varphi \left(T,H\right)-\frac{Nm}{\mu \left(T,H\right)}\left(1-\frac{Nm}{K\left(T,R,H\right)}\right)-\left(a\left(T\right)pMI\left(T\right)\frac{Ih}{Nh}+\mu \left(T,H\right)\right)Sm$$

(Equation 15) $$\frac{dEm}{dt}=a\left(T\right)pMI\left(T\right)\frac{Ih}{Nh}Sm-\left(PDR\left(T\right)+\mu \left(T,H\right)\right)Em$$

(Equation 16) $$\frac{\mathrm{dIm}}{dt}=PDR\left(T\right)Em-\mu \left(T,H\right)Im$$

(Equation 17) φ(T,H)=EFD(T)pEA(T)MDR(T)

The human population (*Nh*) is separated into susceptible (*Sh*), exposed (*Eh*), infectious (*Ih*), and recovered (*Rh*) as:

(Equation 18) $$\frac{dSh}{dt}=-a\left(T\right)b\left(T\right)\left(1-\varepsilon_{r}r\right)\frac{Im}{Nm}Sh+\left(BR-DR\right)S^{H}+\left(Nh-Sh\right)ie$$

(Equation 19) $$\frac{dEh}{dt}=a\left(T\right)b\left(T\right)\left(1-\varepsilon_{r}r\right)\frac{Im}{Nm}Sh-\left(\delta +DR+ie\right)Eh$$

(Equation 20) $$\frac{dIh}{dt}=\delta Eh-\left(\eta +DR+ie\right)Ih$$

(Equation 21) $$\frac{dRh}{dt}=\eta Ih-\left(DR+ie\right)Rh$$

We used *Sm* = 0.22, *Em* = 0.29, *Im* = 0.49, *Sh* = 0.55, *Eh* = 0.05, *Ih* = 0, and *Rh* = 0.40 as the initial proportion of vector and humans in each model compartment, as in.^51^ We set the ratio of vectors to humans to two based on,^34^ with an initial human and vector population equal to 5000 and 10000, respectively.

The **non-climate-dependent** model parameters include the intrinsic incubation period (δ = 5.9 days), human infectivity period (η = −5 days), birth rate (BR, 31.782 per 1000 people per year^51^), death rate (DR, 5.284 per 1000 people per year^51^), and the mosquitoes repellent efficiency (*ε*_r_=0.8).

The repellent use *r* at time *t* is assessed as:

(Equation 22) $$r_{t}=\left\{ \begin{array}{c} r_{\min}\;\text{if}\;\frac{dA^{Dr}}{dt}>\frac{dA^{De}}{dt}\\ A^{De}\;\text{if}\;\frac{dA^{Dr}}{dt}<\frac{dA^{De}}{dt} \end{array}\right.$$

With *r*_min_ as the minimum repellent use assumed equal to 0.2. We assumed that higher variation in drought awareness would lead to lower repellent use, whereas higher variation in dengue awareness would lead to higher repellent use. The immigration/emigration rate at time *t* is assessed as a function of drought migration, as we assumed that a society highly affected by drought may tend to reallocate to an area with more water availability^87^^,^^88^^,^^89^:

(Equation 23) $${ie}_{t}=k_{ie}\left(1-A_{t}^{Dr}\right)$$

With *k*_ie_ as the immigration/emigration constant equal to 0.01.

**The climate-dependent** model parameters are reported in the supplemental information (Table S1). These models were developed by^34^ based on mosquito life-history traits and viral-development rates, as determined by thermal response curves derived from laboratory experiments.

The mosquito mortality rate *μ* is assessed as a function of the temperature and humidity by fitting a spline model based on a pooled survival analysis of Aedes aegypti.^86^

(Equation 24) $$\frac{dRh}{dt}=\left\{ \begin{array}{c} \frac{1}{c\left(T-T_{0}\right)\left(T-Tm\right)}+\left(1-\left(0.01+2.01H\right)\right)0.005\;if\;H<1\;\\ \frac{1}{c\left(T-T_{0}\right)\left(T-Tm\right)}+\left(1-\left(1.22+0.27H\right)\right)0.01\;if\;H\geq 1 \end{array}\right.$$

where the *c*, *T*_*0*_, and *Tm* are the rate constant, minimum temperature, and maximum temperature equal −1.24, 16.63, and 31.85, respectively. Previous research suggested that the saturation vapour pressure deficit *H* is a more informative measure of the effect of humidity on mosquito survival compared with relative humidity.^86^ We calculated *H* as a function of the temperature *T* and relative humidity *RH* in kPa as:

(Equation 25) $$H=6.112\;\exp\left(\frac{17.67T}{T+243.5}\right)\left(1-\frac{RH}{100}\right)\frac{1}{10}$$

Finally, the adult mosquito-carrying capacity *K* is modelled as a modified Arrhenius equation^34^^,^^90^:

(Equation 26) $$K=\frac{EFD\left(T_{0}\right)pEA\left(T_{0}\right)MDR\left(T_{0}\right)\mu {\left(T_{0},H_{0}\right)}^{-1}-\mu {\left(T_{0},H_{0}\right)}^{-1}}{EFD\left(T_{0}\right)pEA\left(T_{0}\right)MDR\left(T_{0}\right)\mu {\left(T_{0},H_{0}\right)}^{-1}}\;{Nm}_{\mathrm{MAX}}\cdot e^{\frac{-EA{\left(T-T_{0}\right)}^{2}}{k_{B}\left(T+273\right)\left(T_{0}+273\right)}}\cdot f\left(R\right)$$

where *N*m_,MAX_ set to the maximum possible mosquito abundance in a population (in this study assumed as twice the human population size following,^34^ *k*_B_ is the Boltzmann constant (8.617 × 10−5 eV/K) EA is the activation energy (0.05),^34^ and *f*(*R*) is the function representing the effect of rainfall on carrying capacity and the availability of fresh water from the rainwater harvesting tank, assessed as:

(Equation 27) $$f\left(R\right)=c\cdot R\cdot \left(R-R_{\min}\right)\sqrt{\left(R_{\max}-R\right)}\cdot \left(1+k^{T,Hab}\frac{V^{T}}{V^{T,\mathrm{MAX}}}\right)$$

where *R*_min_ and *R*_max_ are the minimum rainfall and the maximum rainfall set as 1 mm and 123 mm based on the high probability of flushing,^10^ *c* is the rate constant of 7.86e−5., while our *k*^T,Hab^ represents how strongly household tank water contributes to mosquito breeding habitat in the model, considered 1 in our model. The multiplicative term function of *V*^T^ represents the increase in the availability and persistence of aquatic habitat provided by larger rainwater-harvesting tank volumes. Empirical studies show that Aedes Aegypti pupal productivity is positively associated with tank water volumes and that larger-capacity tanks produce disproportionately more pupae, motivating a monotonic scaling of carrying capacity with stored-water volume.^91^^,^^92^

We built the model considering that precipitation and water availability from the rainwater harvesting tanks strongly affect mosquito populations due to the availability of standing water where eggs are laid and larvae develop, as well as the higher recruitment of mosquitoes that can increase the number of mosquitoes available to bite.

#### Sensitivity analysis

To assess the sensitivity of model outcomes to parametric uncertainty, we performed a global sensitivity and uncertainty analysis using a Latin Hypercube Sampling (LHS) ensemble with 5000 model realizations. A set of key parameters spanning hydrologic operations (e.g., hedging release policy coefficient *K*), drought and dengue behaviours (e.g., awareness decay rates *μ*_Dr_ and *μ*_De_), household storage dynamics (e.g., tank growth rate *ε*_V_, maximum tank capacity *V*^T,MAX^), DENF protection (minimum repellent use *r*_min_, repellent efficacy *ε*_r_), and vector ecology (e.g., mosquito biting rates, infectiousness, carrying capacity *N*_m,Max_ and rainfall–habitat scaling) were varied of ±20% of their assigned parameter value. For each LHS draw, the model was simulated under the three risk behaviours and two model outputs were extracted: (i) the maximum number of infected humans during the simulation (peak *Ih*) and (ii) the mean water shortages value. Global sensitivity was quantified using Partial Rank Correlation Coefficients (PRCC), computed between each sampled parameter and each outcome while controlling for all other parameters. Parameters and outcomes were rank-transformed before computing PRCC to reduce sensitivity to non-normality and nonlinearity and to capture monotonic effects. Uncertainty analysis was based on the same ensemble, summarising outcome distributions across the LHS runs for each risk behaviour, thereby propagating parametric uncertainty through the coupled socio-hydro-epidemiological dynamics (Figures S3 and S4).

### Quantification and statistical analysis

All analyses and visualisations were performed in MATLAB, version R2024a. There are no statistical analyses to include in this study.
