# Supplementary material for: A socio-hydro-epidemiological model for simulating trade-off between dengue infections, water shortages, and adaptive behavior
Source: iScience. 2026 Jun 8;29(6):116310. doi: 10.1016/j.isci.2026.116310 (PMC13264125; doi:10.1016/j.isci.2026.116310)
Supplement: Document S1. Figures S1–S5 and Table S1 [file mmc1.pdf]

iScience, Volume *29*

Supplemental information

A socio-hydro-epidemiological model for simulating trade-off between dengue infections, water shortages, and adaptive behavior

Maurizio Mazzoleni, Francesco Defilippo, Carlo Torti, Eugenia Quiros-Roldan, and Elena Raffetti

Supplementary Figures

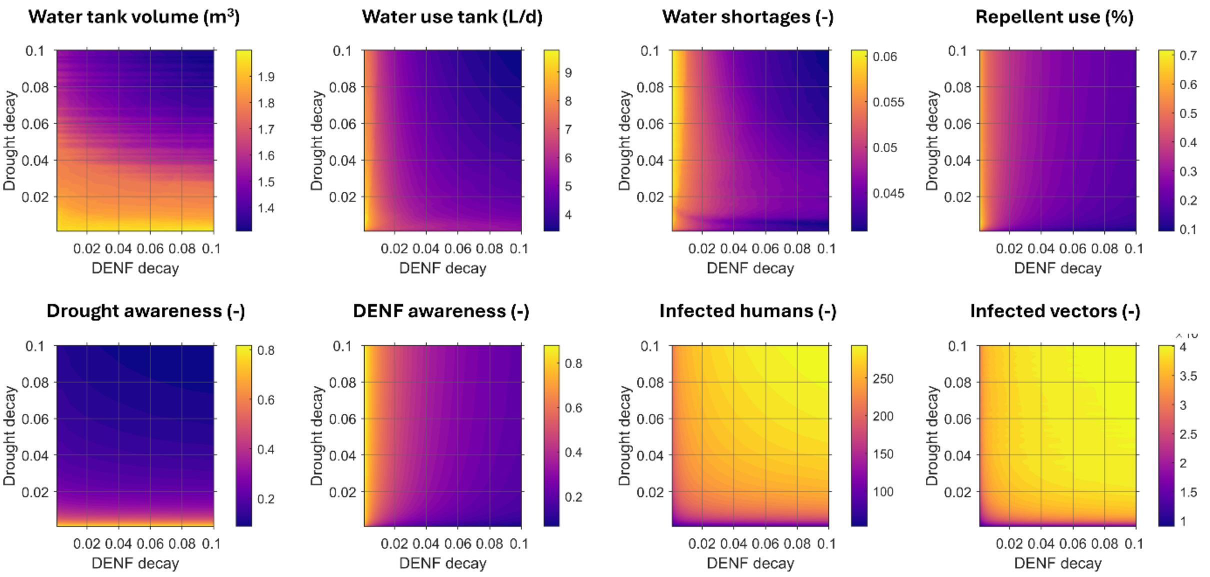

Figure S1. Sensitivity analysis for awareness decay, flood climate scenario. Average value of different model results for different combinations of drought and DENF awareness decay in the case of flood-dominant climate scenario

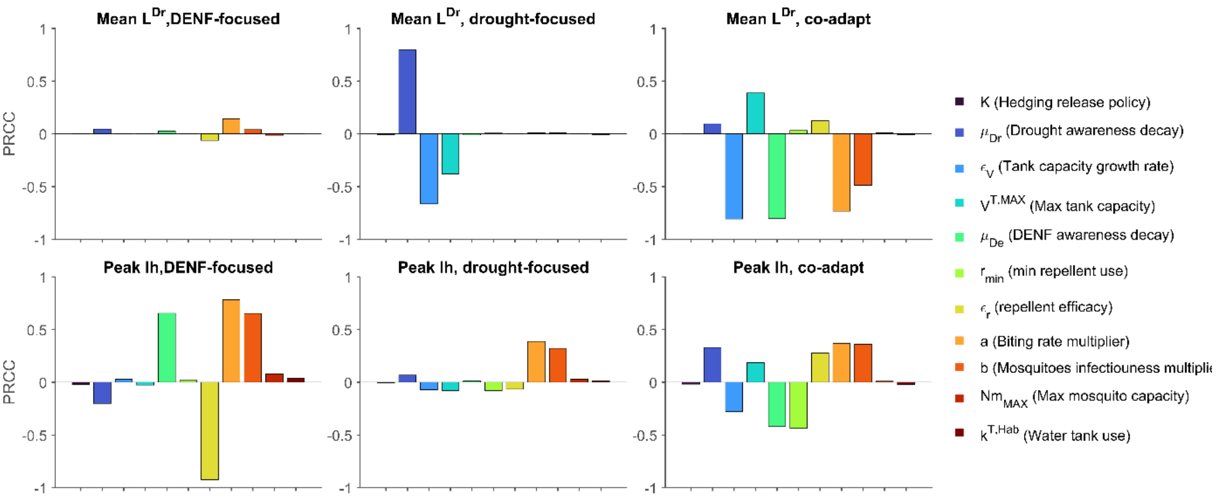

Figure S2. Sensitivity analysis for model parameters, flood climate scenario. Results of the model sensitivity analysis with L^{Dr} water shortages and Ih number of infected humans in case of flood climate scenario

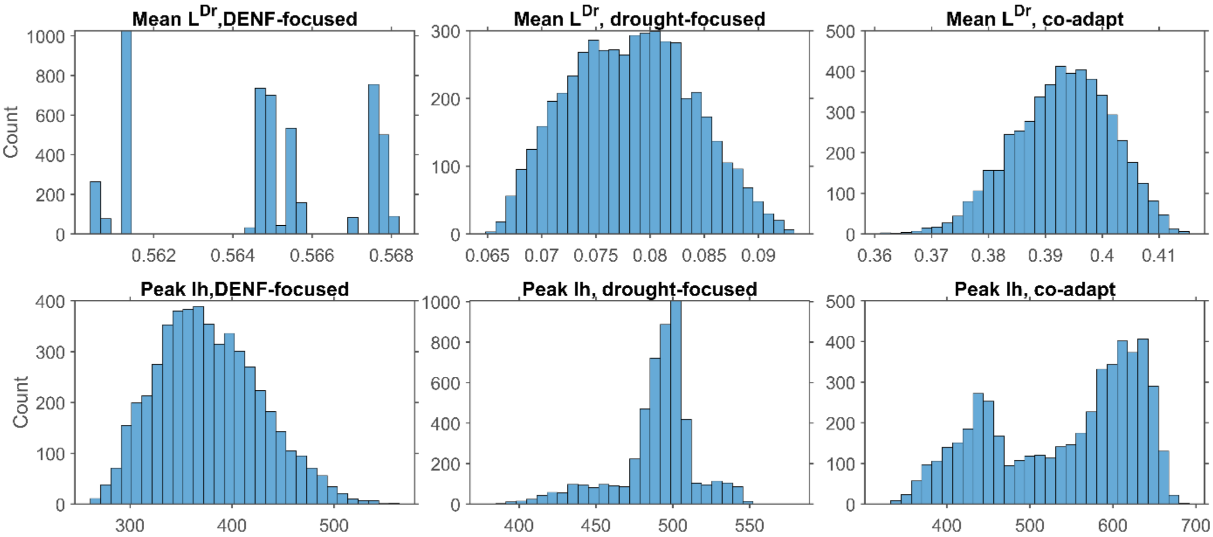

Figure S3. Uncertainty analysis for drought scenario. Results of the model uncertainty analysis with L^{Dr} water shortages and Ih number of infected humans in case of drought climate scenario

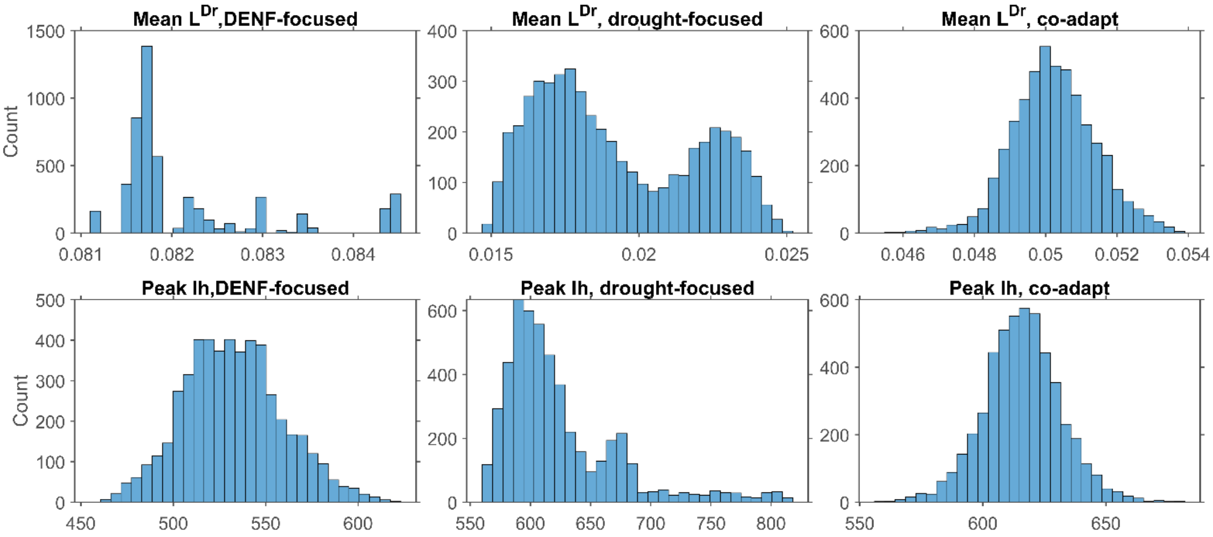

Figure S4. Uncertainty analysis for flood scenario. Results of the model uncertainty analysis with L^{Dr} water shortages and Ih number of infected humans in case of flood climate scenario

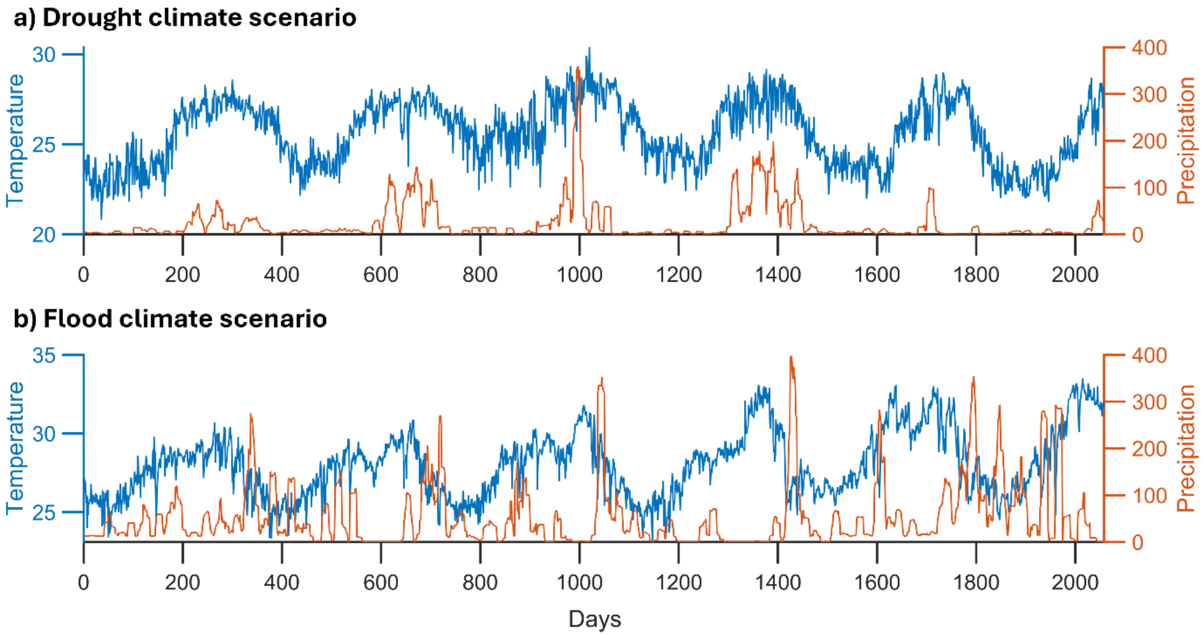

Figure S5. Climate scenarios. Time series of precipitation and temperature for the climate scenarios characterised by the prevalence of drought (a) and flood (b) periods

Supplementary Tables

Table S1. Epidemiological model parameters. Model parameters fit to a Brière [$cT(T-T0)(Tm-T)^{1/2}$] or a quadratic [$c(T- Tm)(T-T0)$] function, where T represents temperature. T_0 and Tm are the critical thermal minimum and maximum, respectively, and c is the rate constant. Thermal responses were fit by [91] and also used in [34]

	**Definition**	**Function**	**c**	**T_0**	**Tm**
a	Biting rate (day−1)	Brière	2.02e−04	13.35	40.08
EFD	Eggs laid per female per day	Brière	8.56e−03	14.58	34.61
pEA	Probability of mosquito egg-to-adult survival	Quadratic	-5.99e−03	13.56	38.29
MDR	Mosquito egg-to-adult development rate (day−1)	Brière	7.86e−05	11.36	39.17
Lf	Adult mosquito lifespan (days)	Quadratic	-1.48e−01	9.16	37.73
b	Probability of mosquito infectiousness	Brière	8.49e−04	17.05	35.83
pMI	Probability of mosquito infection per bite on an infectious host	Brière	4.91e−04	12.22	37.46
PDR	Parasite development rate (day−1)	Brière	6.56e−05	10.68	45.90
